# Service Function Chaining Based on Segment Routing Using P4 and SR-IOV (P4-SFC)

**DOI:** 10.1007/978-3-030-59851-8_19

**Published:** 2020-09-15

**Authors:** Andreas Stockmayer, Stephan Hinselmann, Marco Häberle, Michael Menth

**Affiliations:** 8 University of Tennessee at Knoxville, Knowville, TN USA; 9 Department of Mathematics, KIT für Technologie Karlsruhe, Karlsruhe, Baden-Württemberg Germany; 10 Computational Science, Helmholtz-Zentrum Dresden Rossendorf, Dresden, Sachsen Germany; 11 Extreme Computing Research Center, King Abdullah University of Science and Technology, Thuwal, Saudi Arabia; Chair of Communication Networks, University of Tuebingen, Tuebingen, Germany

## Abstract

In this paper we describe P4-SFC to support service function chaining (SFC) based on a single P4-capable switch and off-the-shelf components. It utilizes MPLS-based segment routing for traffic forwarding in the network and SR-IOV for efficient packet handling on hosts. We describe the P4-SFC architecture and demonstrate its feasibility by a prototype using the Tofino Edgecore Wedge 100BF-32X as P4 switch. Performance test show that L2 throughput for VNFs on a host is significantly larger when connected via SR-IOV with the host’s network interface card instead of over a software switch.

## Introduction

Packet processing at the network ingress or egress typically requires network functions (NFs) such as firewalls, IDS/IPS, NAT, and others. In the past, these functions have been provided as hardware appliances. To reduce costs, many of them are today implemented as applications running on standard server hardware as so-called virtual NFs (VNFs). Complex services may be composed of multiple VNFs. This is called service function chaining (SFC). As the VNFs of a SFC are generally located on different hosts, SFC requires forwarding support in the network. That means, a packet which is classified for a specific SFC must be forwarded along a path that visits all VNFs of the respective SFC in a predefined order.

The IETF has proposed various approaches for this problem. One approach requires per-SFC state in the network, the other requires the ability of a node – we call it the SFC ingress node – to encode a source route in the packet header. Segment routing based on MPLS is one preferred option for source route encoding. It requires the SFC ingress node to push a stack of MPLS labels, but intermediate SFC forwarders just need to pop individual labels. While the latter is simple, pushing a large header stack is hardly supported by today’s affordable hardware.

In recent years, programmable data planes have been developed with the goal to facilitate new headers and forwarding behavior on software and hardware switches. P4 is a widespread programming language to specify switch behavior. It utilizes match-and-action tables for packet forwarding which are populated by a controller. We leverage this technology in this work and refer to [4] for further background.

The contribution of this paper is a simple architecture (P4-SFC) using MPLS-based segment routing for SFC forwarding, a P4-programmable switch as SFC ingress node, and communication with VNFs using SR-IOV hardware virtualization on hosts. To demonstrate the feasibility of P4-SFC, a prototype of P4-SFC is implemented using the Tofino Edgecore Wedge 100BF-32X as hardware platform. It forwards packets at a speed of 100 Gb/s and pushes large header stacks in line speed. Apart from the P4 switch, the prototype consists only of commodity hardware and comprises an SFC orchestrator that allows customers to submit their own VNF implementations that do not need to be SFC-aware.

The paper is structured as follows. In Sect. 2, we give an overview of segment routing, SFC-supporting protocols, and summarize SFC-related activities. Section 3 presents P4-SFC including the P4 pipeline of the SFC ingress node and an P4-SFC orchestrator. Section 4 reports on a prototype implementation of P4-SFC and experiments for validation purposes. Section 5 discusses some performance issues and compares the L2 throughput of VNFs with and without SR-IOV. Section 6 summarizes the work and gives conclusions.

## Related Work

In this section, we first explain segment routing, then we give an overview of existing protocol stacks for SFC, and summarize selected SFC-related activities.

### Segment Routing

With label switching, an MPLS label identifies a connection. The ingress label switching router (LSR) pushes a label onto a packet, intermediate LSRs switch the label according to their forwarding tables, and the egress LSR pops the label. Segment routing (SR) is a new approach for source routing and may leverage MPLS forwarding. Here, a label identifies a segment, which may be a link, a path, a node, etc. The ingress LSR pushes a label stack onto a packet. LSRs forward the packet according to the topmost label and possibly pop it. Thus, with SR, the network can remain unaware of individual connections as only ingress LSRs need to know them to push the right label stack. However, most MPLS nodes can push only few labels. In this work, we utilize SR and program a P4-capable switch for pushing large label stacks.

### Protocol Stacks for SFC

The IETF has identified SFC as a problem for traditional networks due to their topological dependencies [16]. Traditional networks have a rather static configuration but SFC requires a highly dynamical network limiting high availability and scaleability.

A major result of te IETF’s SFC working group is the network service header (NSH) [17]. It consists of three parts: a base header providing information about the header structure and the payload protocol, a service path header containing the path identification and location within a service path, and a context header for metadata.

Another document proposes an MPLS-based forwarding plane for SFC [9]. It suggests tuples consisting of an “SFC context label” and an “SF label” similar to the NSH. The context label identifies the SFC by the contained service path identifier (SPI), and the SF label identifies the next service function to be actioned. In case of label switching, the context label is maintained and used by LSRs to switch consecutive SF labels for VNFs. In case of segment routing, tuples of context/SF labels are stacked by the ingress LSR and are consecutively popped with completed VNF operations. A similar approach is described in another working group draft [7].

These protocols are partly competing and not fully compatible. In all proposed protocol suites, all devices involved in an SFC, e.g., forwarding nodes and NFs, need to be SFC-aware, i.e., they need to respect protocol specifics. P4-SFC allows customers to use VNFs that are not SFC-aware. Furthermore, it leaves the network unaware of SFCs, utilizes only common MPLS labels, and requires forwarding nodes to pop only single labels, i.e., no special hardware features are needed. Only the SFC ingress node pushes a label stack.

### Selected SFC-Related Activities

The ETSI has published a set of documents describing an architecture for networking operations and orchestration (MANO) of NFVs [8]. It provides an overview with focus on interoperability, but it does not offer an NFV/SFC networking stack.

The Open Platform for NFV (OPNFV) [2] has been started by the Linux Foundation in 2014. It is a cooperative project among 20 companies with the goal to develop an NFV infrastructure (NFVI) software stack to build and test NFV functionality. Its long-term goal is to provide a standard platform for NFVI.

Most commercial cloud operators, e.g., Amazon [3] or Microsoft [15], offer configurable, complex services to their customers based on NFV/SFC. Examples of such NFs are firewalls, gateways, and load balancers. These services are comfortable for customers but are limited to functions provided by the cloud operators. P4-SFC allows customers to upload their own VNF binaries.

NFVnice [14] is a user-space scheduler on a host that decides whether a packet is delivered to its desired VNF. It also monitors all VNFs in the system. If VNFs in a later stage of an SFC are overloaded, NFVnice drops packets already at an early stage of the SFC to reduce wasted work.

P4NFV [10] and P4SC [6] propose to implement NFs based on P4-capable hardware because general purpose hardware is too slow for fast packet processing. Their contribution is an architecture for the management of VNFs on P4 switches. The work is applicable to P4-based hardware and software switches.

**Fig. 1.**
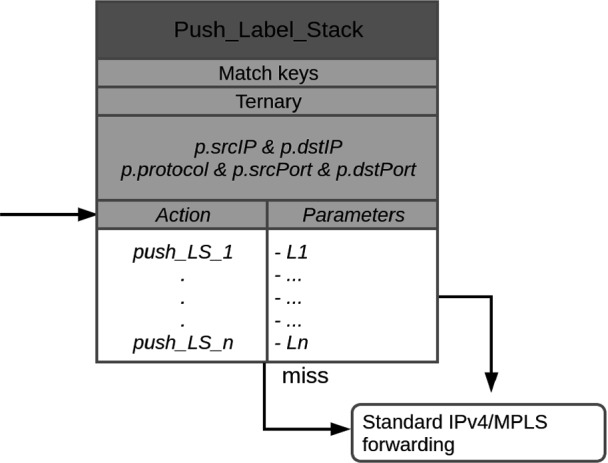
Match-and-action table “push_Label_Stack” to push label stacks of different size.

## Architecture of P4-SFC

In this section we explain the implementation of the P4-based SFC ingress node and how VNFs are efficiently integrated on hosts so that they remain SFC-unaware and transparent for routing.

### Implementation of the SFC Ingress Node

We briefly describe the requirements of the SFC ingress node and explain a P4 pipeline for implementation of this functionality in P4.

**Requirements.** In P4-SFC, the SFC ingress node classifies traffic and adds appropriate MPLS label stacks to packets that require processing by a specific SFC. The classifier identifies flows for a specific SFC. We utilize flow descriptors consisting of source and destination IP addresses, port numbers, and IP protocol number for that purpose. Wildcards are supported. That label stack encodes both the forwarding in the network and the identification of the VNFs. Therefore, the label stack can be large. To keep things simple, we support up to $n=10$ labels in our small testbed (see Sect. 4). However when using jumbo frames, large numbers of labels are possible.

**P4 Pipeline.** We describe the supported header stacks, the ingress and egress control flow, and the  control block in more detail.

*Supported Header Stacks.* Incoming packets are parsed so that their header values can be accessed within the P4 pipeline. To that end, we define up to *n* MPLS labels, an IP header, and a TCP/UDP header.

*Ingress and Egress Control Flow.* The ingress control flow consists of a  control block and an  control block. The  control block adds an appropriate label stack to the packet, i.e., it serves as classifier. The  control block performs simple IP/MPLS forwarding. The egress flow just sends the packet and does not implement any special control blocks.

*Implementation of the*  *Control Block.* The implementation challenge is that an arbitrary number of up to *n* labels need to be pushed. Header sizes are fixed. An intuitive approach is pushing a single label per pipeline execution and recirculating the packet for another pipeline execution until the desired label stack is fully pushed. The drawback of this approach is that pushing *n* labels requires *n*-fold packet processing capacity, which reduces the throughput of the SFC ingress node.

Our solution uses the match-and-action table (MAT) push_Label_Stack whose structure is given in Fig. 1. The MAT utilizes the fields of the source and destination addresses and port numbers as well as the IP protocol number as match keys. A ternary match is used so that wildcards are supported. We provide actions  to push a stack of *i* labels onto the packet. This action has *i* parameters but the table has *n* label entries (L1, ..., Ln). In case of a match, the corresponding action is executed with the appropriate number of arguments. Afterwards, the  control block is carried out. For the implementation of the  control block we reuse available demo code.

### Transparent and Efficient VNF Integration on Hosts

**Fig. 2.**
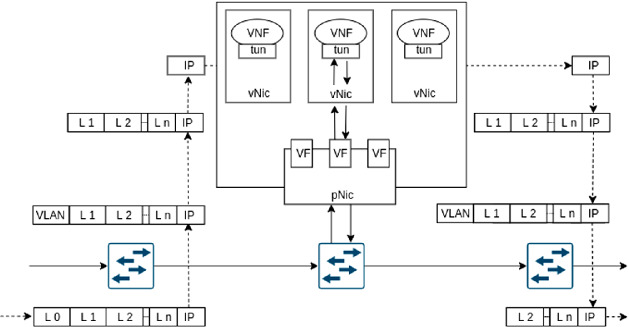
P4-SFC utilizes label stacks in packets for segment routing in the network, but passes only IP packets to VNFs. Therefore, VNF remain unaware of the SFC.

We now specify how packets are forwarded from a switch to a VNF on a host and back. This is challenging since the VNF should remain unaware of the label stack, and the packet forwarding from the host’s network interface card (NIC) to the VM hosting the VNF should be efficient. The following steps are illustrated by Fig. 2.

We assume that up to *N* VNFs are supported by a host, either within a container or a separate VM. Each potential VNF constitutes a logical network segment while the corresponding physical network segment is the switch over which the VNF is reachable. The forwarding table of the switch is configured such that an incoming packet with a topmost label pointing at a specific VNF is equipped with a VLAN tag pointing at the VNF and forwarded to the respective host.

The NIC of the host is statically configured to map packets with VLAN tags to virtual PCI devices that serve as virtual NICs (vNICs) for VMs or containers. These features are enabled by virtual machine device queue (VMDq) and single root I/O virtualization (SR-IOV). These technologies are supported by most contemporary NICs and CPUs. With VMDq, a NIC can have multiple internal queues and with SR-IOV, a so-called Physical Function (PF) can be virtualized into Virtual Functions (VFs). A VF can be passed-through as PCI device to a VM or container. We utilize SR-IOV to pass through a queue of the NIC as a VF to a VM/container in order to serve as a vNIC. The NIC used in our prototype provides up to 128 VFs so that up to $N=128$ VNFs can be supported on a host. More powerful NICs providing even more VFs also exist.

Within a VM/container, the forwarding table of the MPLS Router Module in the Linux kernel is utilized to deliver the IP packet to the VNF without the label stack, to store the label stack, to push the label stack again when the packet is returned from the VNF, and to send the packet to the appropriate egress interface. Then, the packet is returned from the host to the switch in the corresponding VLAN. The switch removes the VLAN tag and the label for the next segment.

### P4-SFC Orchestrator

The P4-SFC orchestrator is written in Python and leverages the libvirt and LXD framework for VM/container management. It interacts with administrators for management purposes and with customers for the specification of SFCs. It places VNFs on hosts and computes paths for SFCs, it launches and terminates SFCs, it adds new hosts and migrates VNFs among hosts.

**Administrator/Customer Interaction and SFC Specification.** The orchestrator offers a CLI interface for maintenance, e.g., for adding a new host to the system or moving VNFs.

Customers provide a configuration file in json format with a description of their SFCs. The specification of an SFC includes a flow descriptor, a list of VNFs, their executable binaries, their resource requirements (CPU, RAM, I/O), and information whether they are to be deployed as VMs or containers. Customers may request permanent storage for a VNF, e.g., for logging purposes, so that it has permission to write to shared network storage. The VNF binaries provided by customers are also saved to shared network storage. VNF applications are required to receive and send packets via  , but they can remain unaware of SFCs.

**VNF Placement and Path Calculation.** The orchestrator determines hosts to run VNFs such that resource requirements communicated by the customers are met. Storage is not part of these requirements since shared network storage is used. If resources are not sufficient, VNFs may be migrated or new hosts may be added. While there is an extensive body of literature on VNF placement, our prototype uses only simple algorithms for this task.

The orchestrator knows the network topology. Either the network topology is static like in our prototype or it can be dynamically discovered with protocols like LLDP [11]. Based on this information, the orchestrator computes paths from the SFC ingress node to desired destinations including the VNFs specified by SFCs. The path calculation is performed whenever a forwarding entry for the SFC ingress node needs to be modified.

**Launch and Termination of SFCs.** The orchestrator holds a disk image as template for VMs/containers supporting VNFs. P4-SFC requires an appropriate configuration of the forwarding table of the MPLS Router Module which is initially applied to the template. The template is copied to every host so that VMs/containers can be cloned from it. Resources required by a VNF are provided by the customer’s SFC description and are enforced by the orchestrator using appropriate configuration files for the VM/container. A libvirt xml definition specifies the hardware resources assigned to a VM. Similarly, an LXD configuration file uses cgroup statements to limit the kernel space resources available to the container.

If an SFC is to be launched, the orchestrator determines for each VNF a host with sufficient resources and finds a free VF on the NIC of that host. This VF determines the label for the VNF. The orchestrator defines a VM/container with suitable parameters, i.e., the VM/container template, sufficient resources, the VF, and a pointer to the VNF binary. It then starts the VM/container and the appropriate VNF binary from the shared network storage. Finally, the SFC ingress node is configured. To that end, a path is computed for the SFC and an entry is added to the MAT push_Label_Stack (see Fig. 1) containing the flow descriptor and the label stack for the SFC. The flow descriptor is needed for packet classification.

If an SFC is to be stopped, the VMs/containers with its VNFs are terminated and the corresponding entry is removed from the MAT push_Label_Stack.

**Adding a New Host.** To add a new host to P4-SFC, the orchestrator needs ssh access and permissions for VM/container management on the new host. It initially scans for available resources on the new host and adds them to its pool of available capacities. It then copies the VM/container templates to the host and configures the virtualization frameworks.

To make the new host and its potential VNFs reachable in the network, the forwarding table of the switch to which the host is attached is equipped with forwarding entries for the labels of all potential VNFs on the new host. If a host is removed, the corresponding labels are removed from the switch.

**VNF Migration.** VNFs may need to be migrated to another host, e.g., for maintenance purposes. The orchestrator supports this process by first cloning and starting the VNF on the new host, changing the respective entry of its SFC in the MAT push_Label_Stack, and terminating the VNF on the old host.

## P4-SFC Prototype

We first give an overview of the testbed and describe functional tests. Finally, we report on the virtualization platform of the hosts.

**Fig. 3.**
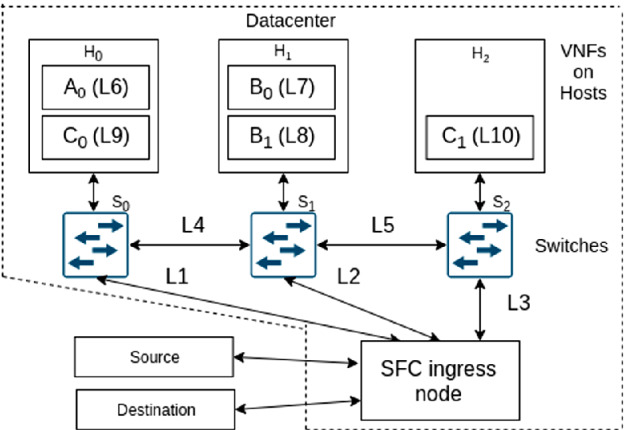
Testbed for functional tests of P4-SFC.

### Testbed

Figure 3 illustrates the testbed setup. A small datacenter which consists of an SFC ingress node, three forwarding nodes, and three servers hosting VNFs. Traffic is forwarded using segment routing using the bidirectional labels associated with the links. The MPLS forwarding nodes are standard Linux PCs with software switches using the mpls_router kernel module and iproute2. The SFC ingress node is a Tofino Edgecore Wedge 100BF-32X with the implementation as reported in Sect. 3.1. In addition, an orchestrator as outlined in Sect. 3.3, but omitted in the figure, controls the testbed.

### Functional Tests

We conduct the following experiments. An external source sends traffic via the datacenter to a destination. Within the datacenter, traffic is treated by an SFC. We experiment with three different SFCs that consist of the SFCs *A*, *B*, and *C*. We run tcpdump on source, destination, and the hosts to observe whether a packets are sent, received, or delivered to a specific VNF. We describe three experiments in which traffic was received by the destination.

SFC *A* contains only the single VNF $A_0$ and the label stack is (*L*1, *L*6). The successful experiment demonstrates that the implemented segment routing works.

SFC *B* contains two VNFs $B_0$ and $B_1$ on the same host and the label stack is (*L*2, *L*7, *L*8). The successful experiment shows that multiple VNFs can be reached on the same host. To that end, the traffic is forwarded from $B_0$ via $S_2$ to $B_1$.

SFC *C* contains two VNFs $C_0$ and $C_1$ on different hosts with the label stack *L*1, *L*9, *L*4, *L*5, *L*10. The successful experiment shows the correct operation of alternating VNF delivery and network forwarding.

### Host Virtualization Platform

We use servers with a Xeon Gold 6134 processor, 8 cores, and 128 GB RAM as hosts. Linux kernel 5.3.10 serves as operating system. We leverage the Intel VT-x feature to enable hardware-accelerated virtualization. We use Linux Kernel-based Virtual Machine (KVM) [13] as a hypervisor. VMs are created based on QEMU and are managed using the libvirt [18] framework. This approach enables almost native performance for VMs [12]. Containers are supported with the LXD [5] framework using cgroups [1] to isolate containers from each other and manage resource allocation.

In contrast to VMs, containers are lightweight so that many of them can be accommodated on a single host. However, isolation among them is not perfect. In addition, there is some risk that malicious VNFs exploit security breaches and compromise the host. This tradeoff influences the choice whether VMs or containers should be used for VNFs. Thus, trusted VNFs can be deployed as containers so that they can benefit from a smaller resource footprint and a faster starting time compared to VMs.

We presented only one specific instantiation of P4-SFC. KVM-based virtualization on host could by easily substituted by e.g. XEN [20] without modification of the orchestrator. Segment routing could be implemented with IPv6 instead of MPLS but this requires changes to the SFC ingress node and the orchestrator.

## Performance Evaluation

We first compare the forwarding efficiency VNF interconnection with SR-IOV and a virtual switch on the host and then we discuss additional performance aspects.

### Performance Comparison

We compare the throughput for a VNF integrated with SR-IOV as presented in Sect. 3.2 with the one of a VNF connected via the Open vSwitch (OVS) software switch [19]. We utilize the same host platform as in Sect. 4.1 and two Mellanox ConnectX-5 NICs with 100 Gb/s. We set up 2 VMs with 8 cores each and utilize both iperf2 and iperf3 for TCP throughput test for 10 s. Iperf2 supports multiple threads, but iperf3 is the newer version. To test SR-IOV-based integration, each of the two VMs uses one VF on different NICs. To test OVS-based integration, both VMs are connected only via the OVS and not via a real NIC, which is an optimistic approximation for OVS-based VNF communication.

**Table 1.** L2 throughput for VNFs with different integration.

L2 packet size	Forwarding technology	iperf3 1 flow	iperf2 1 flow	iperf2 8 flows
1500	OVS	3.11 Gb/s	3.3 Gb/s	3.24 Gb/s
bytes	SR-IOV	32.3 Gb/s	36.3 Gb/s	93.4 Gb/s
104	OVS	54.3 Mb/s	2.24 Mb/s	4.24 Mb/s
bytes	SR-IOV	2.24 Gb/s	2.17 Gb/s	7.64 Gb/s

Table 1 shows the results. With iperf3, multithreading is not supported so that we used it only for experiments with a single flow. For large packets and OVS, around 3 Gb/s L2 throughput are achieved, both with iperf2 and iperf3, with 1 and 8 flows. Thus, 3.3 Gb/s seems to be the upper limit of OVS. With SR-IOV, we obtain a L2 throughput of 32.3 Gb/, 36.3 Gb/s, and 93.4 Gb/s in different experiments. Thus, the L2 throughput is up to 28 times larger than with OVS. For small packets, the L2 throughput is significantly reduced for both OVS and P4-SFC. In addition, we observe TX errors on the OVS NICs. With iperf2 and a single flow we even witness interface resets. Therefore, the throughput is extremely low in those cases. These problems were not observed with SR-IOV.

### Additional Performance Aspects

We discuss additional performance aspects.

**Ingress Node Forwarding Speed.** Tofino performs P4 code in line speed so that its full capacity 100 Gb/s can be used for packet classification and encapsulation with label stacks.

**Encapsulation Overhead.** The segment routing approach used in P4-SFC imposes multiple labels per packet. Each MPLS label is only 4 bytes large. Thus, the header overhead for a stack of 10 labels amounts only to 40 bytes which is the size of a single IPv6 header, which has hardly any impact on forwarding performance.

**Ingress Node Scalability.** The SFC ingress node needs to support a large number of SFCs, therefore, the size of the entries in the MAT push_Label_Stack (see Fig. 1) is critical. Each entry consists of ingress and egress IPv4 addresses (2 $\times$ 4 bytes), ingress and egress ports (2 $\times$ 2 bytes), IPv4 protocol number (1 bytes) for SFC classifier and 10 MLPS labels (10 $\times$ 4 bytes) for segment routing. This amounts to 53 bytes per table entry, which allows for a number of SFCs in an order of magnitude of 100 K.

**Traffic Engineering.** Segment routing has potential for traffic engineering so that a smart orchestrator could optimize network performance.

## Conclusion

In this work, we proposed P4-SFC as an architecture for SFC. It has a P4-based SFC ingress node which can push large header stacks needed for segment routing. It uses SR-IOV-based host virtualization to achieve high VNF throughput. It uses plain MPLS forwarding and allows VNFs to be SFC-unaware and allows customers to upload own binaries as VNFs. P4-SFC has been demonstrated as a prototype. The SFC ingress node has been implemented on the Tofino Edgecore Wedge 100BF-32X. The orchestrator controls both the SFC ingress node and hosts. Experiments showed that the chosen approach for host virtualization is more powerful than using software switches.
